# Continuous Monitoring of Air Purification: A Study on Volatile Organic Compounds in a Gas Cell

**DOI:** 10.3390/s20030934

**Published:** 2020-02-10

**Authors:** Alaa Fathy, Marie Le Pivert, Young Jai Kim, Mame Ousmane Ba, Mazen Erfan, Yasser M. Sabry, Diaa Khalil, Yamin Leprince-Wang, Tarik Bourouina, Martine Gnambodoe-Capochichi

**Affiliations:** 1 ESYCOM Lab, UMR 9007 CNRS, Université Gustave Eiffel, ESIEE Paris, 77454 Marne-la-Vallée, France; alaa.fathy@esiee.fr (A.F.);; 2 Si-Ware Systems, 3 Khalid Ibn Al-Waleed St., Heliopolis, Cairo 11361, Egypt; 3 Faculty of Engineering, Ain-Shams University, 1 Elsarayat St. Abbassia, Cairo 11535, Egypt

**Keywords:** air purification process, MEMS-FTIR spectroscopy, VOC, online analysis, ZnO nanowires array, gas capturing, photocatalysis

## Abstract

Air pollution is one of the major environmental issues that humanity is facing. Considering Indoor Air Quality (IAQ), Volatile Organic Compounds (VOCs) are among the most harmful gases that need to be detected, but also need to be eliminated using air purification technologies. In this work, we tackle both problems simultaneously by introducing an experimental setup enabling continuous measurement of the VOCs by online absorption spectroscopy using a MEMS-based Fourier Transform infrared (FTIR) spectrometer, while those VOCs are continuously eliminated by continuous adsorption and photocatalysis, using zinc oxide nanowires (ZnO-NWs). The proposed setup enabled a preliminary study of the mechanisms involved in the purification process of acetone and toluene, taken as two different VOCs, also typical of those that can be found in tobacco smoke. Our experiments revealed very different behaviors for those two gases. An elimination ratio of 63% in 3 h was achieved for toluene, while it was only 14% for acetone under same conditions. Adsorption to the nanowires appears as the dominant mechanism for the acetone, while photocatalysis is dominant in case of the toluene.

## Introduction

The development of human industrial activities, daily life habits and increasing urbanization are leading to an intensification of environmental pollution, mainly of water and air by chemical agents. These include volatile organic compounds (VOCs) such as aldehydes, ketones, hydrocarbons, aromatics… and semi-volatiles such as polycyclic aromatic hydrocarbons (PAHs); inorganic compounds such as CO, Nox, O_3_ and heavy metals. VOCs are harmful to public health and contribute into climatic changes [1,2]. The issue of air quality becomes an important topic and increases the research to find solutions to the purification of the air we breathe and also water. The aim is to develop innovative, low-cost, selective, environmentally friendly materials capable of capturing and destroying these pollutants, in conjunction with a real time monitoring technology. Such real time monitoring is crucial as it will enable triggering the operation of depollution systems when needed, hence improving their efficiency. From fundamental point-of-view, developing a tool for *in situ* real-time monitoring of the VOCs will contribute to the understanding of the mechanisms involved in the purification process as well as the chemical kinetics when several gases are interacting to each other, a typical situation that is commonly faced with tobacco smoke.

For several decades, nanostructured metal oxide semiconductors such as titanium dioxide TiO_2_ or zinc oxide ZnO have responded to the issue of destruction of the VOCs [3]. Indeed, because of its wide band interval (3.37 eV) and its great excitonic binding energy (60 meV), ZnO has very interesting properties. It can be used in several fields of applications such as energy harvesting [4,5], photovoltaics [6,7], photodetector [8,9], gas sensing [10,11] and photo-catalysis [12,13,14,15,16] ZnO-NWs can be easily obtained by hydrothermal synthesis, a simple and inexpensive method that can be implemented at low temperatures [17,18].

Different instruments are used for the VOCs detection and quantification, such as gas chromatography, mass spectrometers [19], Fourier Transform Infra Red (FTIR) spectrometers [20], tunable diode laser spectrometers (TDLS) [21] and photoacoustic-based TDLS [22]. TDLS and photoacoustic-based TDLS are characterized by high sensitivity and selectivity compared to FTIR spectroscopy, however, FTIR spectroscopy has a much larger spectral range which means the capability of measurement of a wide variety of different gases at the same time. Photoacoustic-based TDLS is characterized by independence of the sensitivity on gas path length allowing to use small gas cells of a few cm in length [23] and further miniaturization of the whole system using semiconductor technology [24]. Using Microelectromechanical systems (MEMS) technology, one could have a miniaturized gas sensor based on the FTIR principle, leading to a compact and cheap on-site and timely measurement [25]. Such MEMS-based FTIR spectrometer already succeeded in measuring C_2_H_2_ with a detection limit of 350 ppm and CO_2_ with a detection limit of 1800 ppm using a gas cell of 10 cm length [26]. The use of a multi-pass gas cells with the MEMS FTIR spectrometer and high brightness light source has been reported for improving the detection limits [27]. In [28], a FTIR spectrometer based on using a translation MEMS mirror with other discrete optical components was used to measure propane and butane using a path length of 6 cm. Gas measurement was performed using a MEMS lamellar grating FTIR spectrometer in [29]. Moreover, a MEMS-based tunable filter and a swept laser source were also used to measure CO and CO_2_ [30,31].

**Figure 1:**
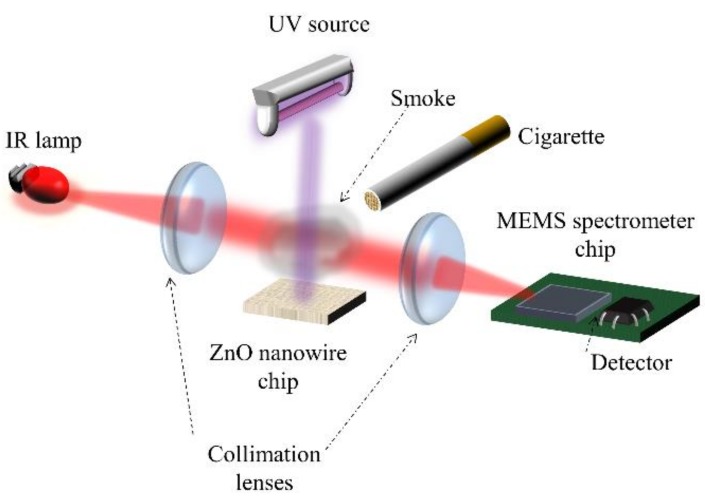
Photocatalysis setup with timely monitoring of air purification.

In this work, we focused our study on the demonstration of the photocatalytic effect of ZnO-NWs on the degradation reactions of the polluting gases contained in tobacco smoke, in order to evaluate its efficiency in the purification of the indoor air. For the study of simple gases contained in tobacco smoke, acetone and toluene are chosen as models. We aim to study the efficiency of ZnO-NWs of these pollutants, by distinguishing the contributions of the two main mechanisms involved, that is, adsorption and photocatalysis. Therefore, both capturing and photocatalysis reactions were continuously tracked by using a new hand-held FTIR spectrometer (Neospectra^®^, Si-Ware Systems, Heliopolis, Egypt) operating in the mid-infrared (MIR) spectral range. The setup for continuous monitoring of the depollution process is depicted in Figure 1.

## 2. Materials and Methods

### 2.1. ZnO-NWs Synthesis and Characterization

**Figure 2:**
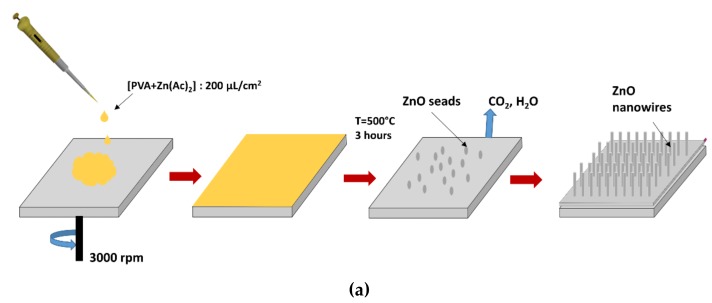

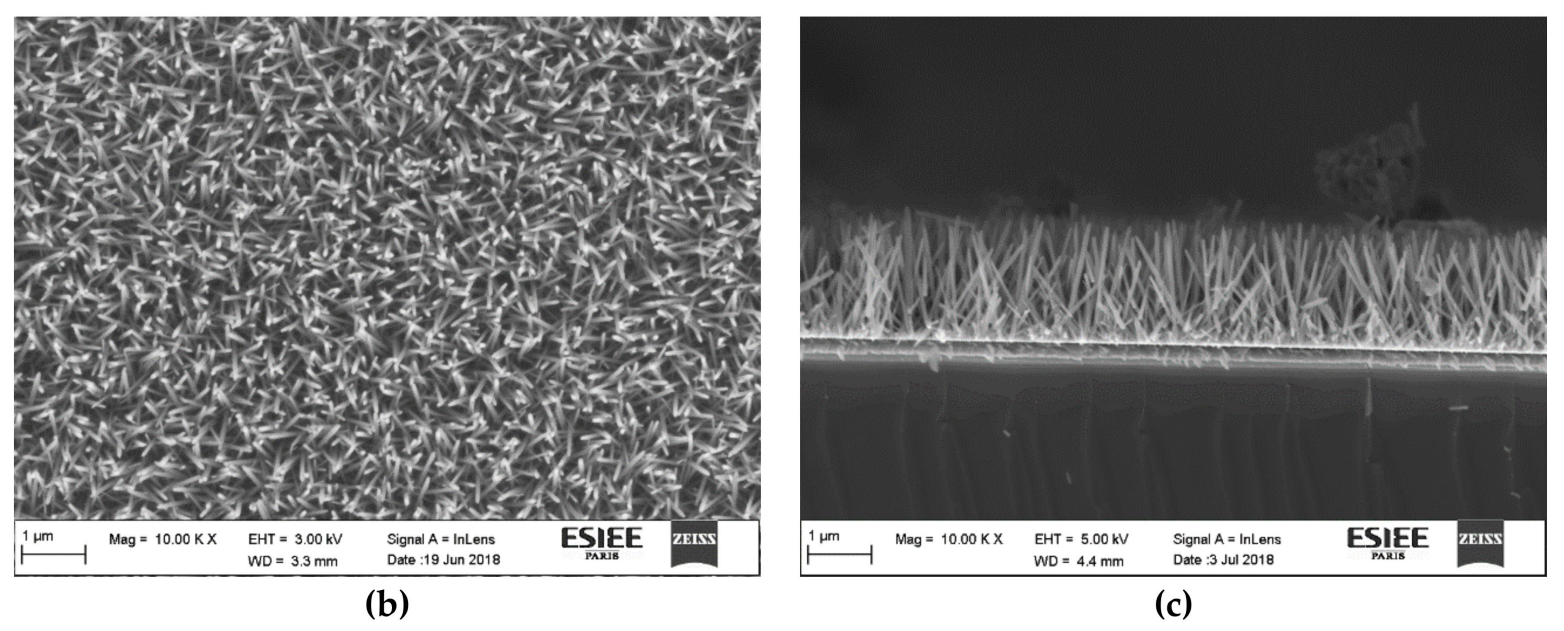
(**a**) Steps of ZnO-NWs synthesis; (**b**) Top view and (**c**) Cross Section SEM images of ZnO-NWs.

The ZnO nanowire arrays were synthesized by using two-step hydrothermal method. Chemical reagents (Sigma Aldrich, Saint-Quentin-Fallavier, France) for synthesis were used. A buffer solution is obtained by mixing 2 mg of polyvinyl alcohol (PVA) and 0.25 mg of zinc acetate dihydrate [(Zn (CHCOO)_2_∙2H_2_O) in 25 mL of DI water. The synthesis steps are shown in Figure 2a. In the first step, after meticulous cleaning of the surface of the silicon wafer (Si-Wafer), the buffer layer was spin-coated and then annealed at 500 °C during 3 hours in order to obtain ZnO nucleation seeds according to the protocol described in our previous work [15]. For the second step, an equimolar aqueous growth solution of 25 mM concentration was obtained with zinc nitrate Zn (NO3)_2_; hexahydrate and Hexamethylenetetramine (HMTA). Then, ZnO-NWs were grown directly by hydrothermal synthesis on Si-wafer surface. Hydrothermal growth was carried out in the growth solution for 4 h at 90 °C in Teflon sealed reactor, which contains substrate covered by ZnO nucleation seeds. The ZnO-NWs morphology was investigated by SEM (FEG-SEM, NEON 40 ZEISS, Jena, Germany) operating at 10 kV. SEM observations showed a quite homogeneous morphology under optimized elaboration conditions. The cross-section SEM images allowed us to determine an average length of 1.8 ± 0.1 µm with an average diameter of 51 ± 5 nm. The captured images are shown in Figure 2b,c.

### 2.2. Pollutants Degradation by Adsorption and Photocatalysis

The adsorption phenomenon is a physisorption reaction, which takes place at temperatures below 200 °C; adsorption is non-destructive and reversible. The reaction takes place on the surface and does not affect the intrinsic nature of the semiconductor material. It depends on the nature of the host material and the target gas.

**Figure 3:**
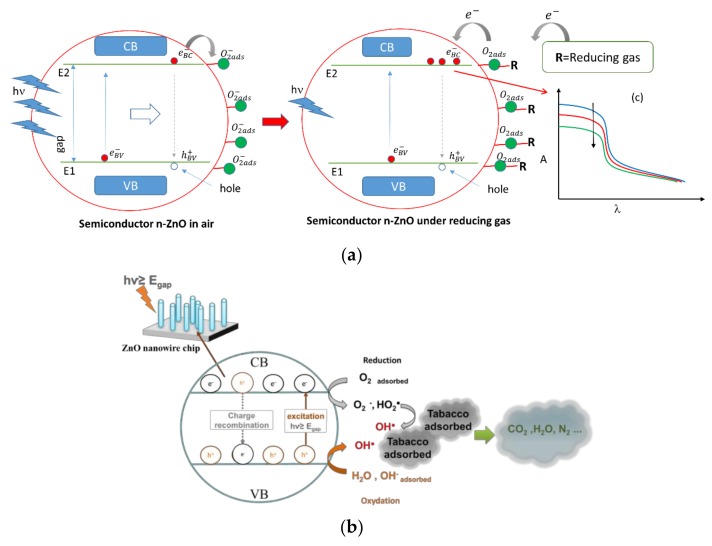
(**a**) Process of air purification by adsorption using ZnO-NWs, (**b**) Process of air purification by photocatalysis using ZnO-NWs under UV light exposure.

In the case of characterization by UV optical absorption spectroscopy, the optical response depends on the densities of electron states in the valence band and holes in the conduction band of ZnO. In fact, for a photon hν, the energy states E1 and E2 of the electrons are conditioned by the relationship: hν = E2 − E1. Therefore, to observe an absorption phenomenon of an energy photon hν, it is necessary to have an electron of energy E1 in the valence band and an empty state (hole) of energy E2 in the conduction band. In the presence of a reducing gas, the electrons reintroduced into the ZnO conduction band will occupy and block part of the states allowed for the transition E1 → E2. Thus, this will reduce the transition rate, resulting in a decrease in the measured absorbance (Figure 3a).

On the other hand, photocatalytic decontamination is an irreversible process. It is an effective way to degrade and mineralize a wide range of organic pollutants. Therefore, it appears as a promising solution for the purification of the water and the air by simple exposure to the UV light. Photons with an energy greater than or equal to the bandgap of the semiconductor lead to the formation of electron-hole pairs. These photogenerated electron-hole pairs are responsible for the redox reaction with H_2_O and O_2_ previously adsorbed on the semiconductor surface and lead to the formation of hydroxyl radicals which have a strong oxidizing power. On the surface, the hydroxyl radicals would oxidize the pollutants and lead to their degradation and mineralization. The process is described in Figure 3b.

### 2.3. Experimental Setup Using MEMS-based FTIR Spectrometer

A MEMS-based FTIR spectrometer is a low cost, miniaturized spectral sensor operating in both the near infrared (NIR) and mid-infrared (MIR) spectral ranges. This device is a developed version of the Neospectra^®^ MEMS spectrometer. The MEMS spectrometer measures the spectral content of the input light. The Michelson interferometer is implemented on silicon chip, where all optical components are fabricated simultaneously on the same silicon chip and self-aligned by the lithography process. The module used in the experiment has a spectral range extending from 1.6 to 4.9 𝜇m with a spectral resolution down to 33 cm^−1^. The spectrometer was configured to 50 cm^−1^ during the measurements. It is worth mentioning that the two VOCs under consideration (either acetone or toluene) have absorption lines at nearly 3000 cm^−1^, which can be easily discriminated from the absorption lines of other major air contents such as CO_2_ (2350 cm^−1^) and H_2_O (3650−3850 cm^−1^). However, such resolution of 50 cm^−1^ make it difficult –but not impossible- to differentiate between acetone and toluene as both gases absorb lines at nearly 3000 cm^−1^. In our work, it was not our purpose to differentiate between these two VOCs. Indeed, our focus is to study those gases separately.

**Figure 4:**
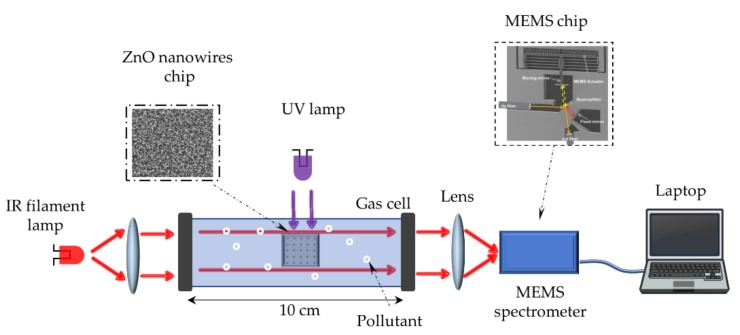
Experimental setup of pollutants purification within the gas cell by photocatalysis on ZnO-NWs chip. The depollution process is continuously monitored using MEMS spectrometer coupled to the gas cell.

The whole setup is presented in Figure 4. The photocatalysis is implemented as a remediation solution to reduce different gas pollutants, such as acetone and toluene. It was conducted inside a direct path gas cell (cell volume is *V_cell_* = 132 mL and path length is 10 cm). The wall of the gas cell is made of Pyrex while two potassium bromide (KBr) windows were used for coupling the IR light in and out. First, background measurement was taken with gas cell containing pure air, taken as a reference while the ZnO-NWs sample (1.5 cm × 1.5 cm) is inserted inside the cell. After that, pollutants were added inside the cell and the new spectrum is recorded. By adding a controlled volume of pollutant in the liquid state, its volatility leads to a well-controlled concentration in the gas state after complete evaporation. After that, the pollutants are introduced inside the cell by heating the whole cell. For acetone, *V_liquid_* = 20 µL are used and this corresponds to a concentration around 5% which can be calculated from the following equation:

(1) $$C\;\left(ppm\right)=\frac{P_{gas}}{P_{Total}}=\frac{R\;T\;\rho V_{liquid}}{P_{Total}V_{cell}M_{W}}$$

where *P_gas_* corresponds to partial pressure of pollutant, *P_Total_* is the total pressure inside the gas cell which is equal to 1 atm., *R* is Boltzmann constant, 𝜌 is the pollutant density in liquid state and *M_w_* is molecular weight of pollutant. The cell was irradiated with an UV lamp (reference LC8, 4500 mW/cm², λ = 365 nm, Hamamatsu, Hamamatsu City, Japan) localized above the cell with a distance of 10 cm between the lamp and ZnO-NWs sample. Due to Pyrex absorption, we estimated an intensity of 3263 mW/cm² being received by our ZnO-NWs. After starting the UV illumination, gas pollutant concentration was monitored every 20 min during 3 h, by the Neospectra^®^ MEMS FTIR (Heliolpolis, Egypt). In addition, the same experiment was repeated using 10 µL of toluene which corresponds to a gas concentration of 1.7%.

## 3. Results and Discussion

### 3.1. Acetone Measurements

**Figure 5:**
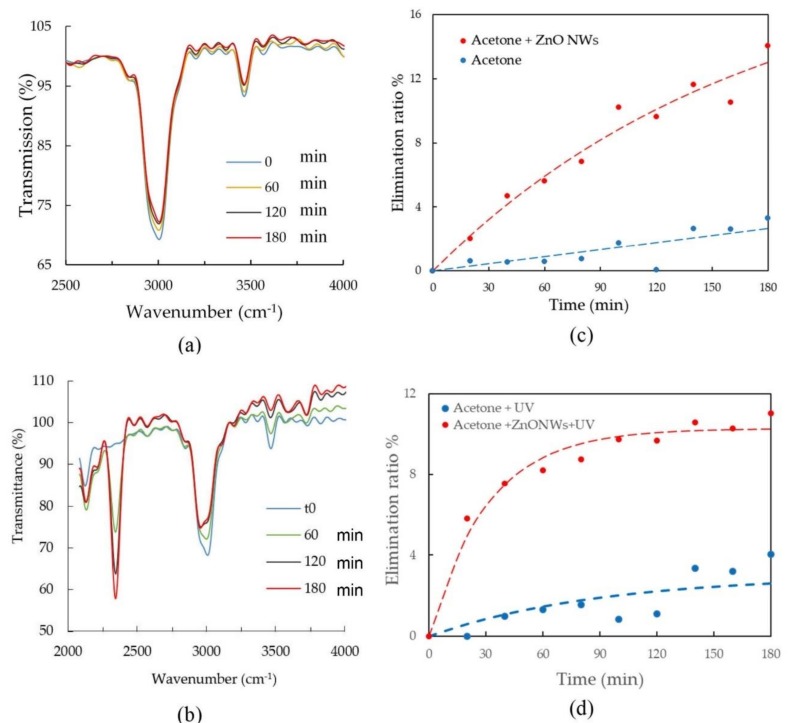
(**a**,**b**) Transmission of acetone measured using MEMS spectrometer at different measurement time (in min.) (**a**) without and (**b**) with the presence of UV. (**c**,**d**) The elimination ratio of acetone versus time in case of absence and presence of ZnO (**c**) without and (**d**) with the presence of UV. Dash lines represent exponential fit ($1-e^{-t/\tau }$) for the measured points, where τ is a time constant.

The transmission of the acetone was measured using the MEMS spectrometer every 20 min. The transmission spectra during the purification process of acetone using ZnO is shown in Figure 5 at different acquisition times. The elimination ratio is also calculated for the four different cases: without/with ZnO chip in the absence/presence of UV light. The elimination ratio at an observation time t is calculated as following expression:

(2) $$E\left(t\right)=100\times \frac{A\left(t_{0}\right)-A\left(t\right)}{A\left(t_{0}\right)}$$

where $A\left(t_{0}\right)$ and $A\left(t\right)$ represent the absorbance at given wavelength at initial time $t_{0}$ and observation time t, respectively. The absorbance value at 2970 cm^−1^, which corresponds to C-H bond, was used to monitor the degradation of acetone with the time. The transmission of the acetone was recorded every 20 min for 3 h. In the absence of the UV, the recorded transmission curves at different sampling times are shown in Figure 5a. 

Figure 5c shows the degradation of the acetone with time when one looks at the decrease of the absorbance around 2970 cm^−1^. The elimination ratio versus time, was calculated in the presence of ZnO and compared to the case of absence of ZnO as depicted in Figure 5c. Using ZnO enhances the elimination ratio during all the observation time. The elimination ratio after 3 hours is around 14% in case of ZnO compared to only 3 % without ZnO, as shown in Figure 5c. This shows an enhancement of the elimination ratio by 4.6 times. Also, returning to Figure 5b, one observes that the ${\mathrm{CO}}_{2}$ absorption line at 2350 cm^−1^ increases with time proving the increase in CO_2_ concentration as a by-product of photo-oxidation process. This is aligned with the theoretical expectations described in Figure 3a.

The experiments are then repeated using UV light. This leads to a photo-oxidation process in the absence of ZnO chips and photo-catalysis in the presence of ZnO chips. The transmission of the acetone at different time are depicted in Figure 5c showing the degradation versus time. The elimination ratio is also plotted in Figure 5d. The enhancement can be seen clearly when using ZnO. For example, after three hours the elimination ratio in case of ZnO was 11% while removing the ZnO chip leads to nearly 0%. Elimination ratio fluctuates around zero in case of the absence of ZnO for measurement time less than two hours, as shown in Figure 5d. This is because the decrease in the concentration of the acetone is within the spectrometer noise/ systematic variation. It is worth mentioning that the use of the UV light leads to a faster elimination (time constant of nearly 30 minutes) at the early stage as shown in Figure 5d, while the elimination ratio is slower without the UV light as shown in Figure 5c, even though the final states reach the same order of magnitude of 10% after 3 h.

### 3.2. Toluene Measurements.

**Figure 6:**
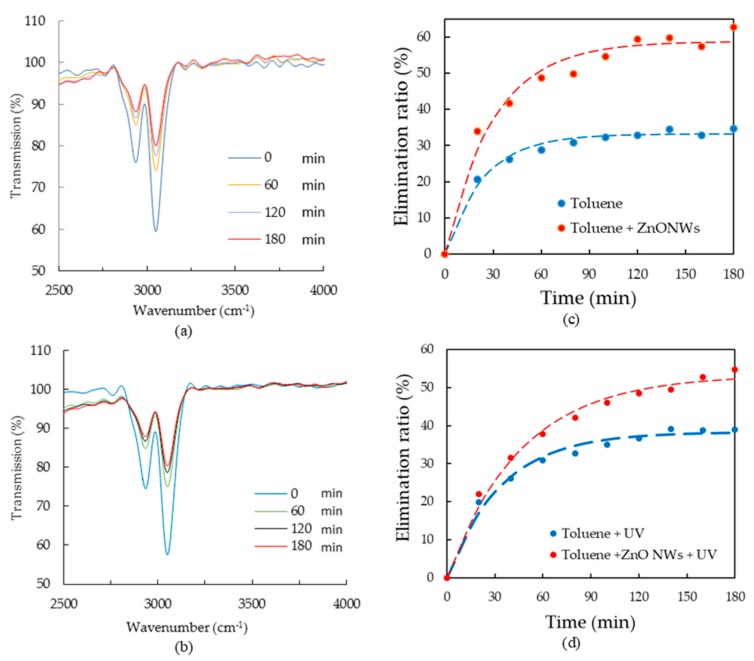
(**a**,**b**) Transmission of toluene measured using the MEMS spectrometer at different measurement time (**a**) without and (**b**) with the presence of UV. (**c**,**d**) The elimination ratio of toluene versus time in case of absence and presence of ZnO (**c**) without and (**d**) with the presence of UV. The elimination ratio is calculated using the gas absorbance at 3042 cm^−1^. Dash lines represent exponential fit for the measured points.

In order to extend the study to different VOCs, a similar experiment has been carried out for toluene degradation. First, the toluene concentration variation is monitored using the MEMS spectrometer without applying the UV light. The transmission spectra measured at different acquisition times are shown in Figure 6a. The elimination ratio is calculated using the absorbance value at 3042 cm^−1^ and shown in Figure 6c. After three hours of adsorption, the elimination ratio reaches around 63% in case of ZnO while it reaches 35% in the absence of ZnO. The elimination ratio is enhanced over all the observation time. In Figure 6b, the transmission curves of toluene in case of applying the UV light are shown. The elimination ratio of the respective process is plotted in Figure 6d. The figure shows an elimination ratio around 55% in case of using ZnO with the UV light compared to 38% without using ZnO with the UV light.

When comparing the experimental results obtained with the two VOCs considered in this work, namely acetone and toluene, it appears that much higher elimination ratios were attained with the toluene (up to 63%) compared to acetone (14%) after 3 hours. However, one can also note that the ZnO improves the purification up to 1.4 times for toluene. It appears that ZnO plays a crucial role in the efficiency of the acetone removal since almost no elimination (nearly 3%) is observed in the absence of ZnO (Figure 5). On the contrary, when considering the removal of the toluene (Figure 6), the presence of ZnO is not as crucial as for acetone since a significant elimination of toluene (35% up to 38 %) is obtained even without ZnO. However, the presence of ZnO for removal of toluene leads to further enhancement of elimination (55% to 63%), in both cases of with and without using UV light.

These results suggest that both adsorption and photocatalysis play a role in the degradation of the toluene. It is also worth-mentioning that elimination of the acetone is also obtained in both cases of using UV light or not, reaching quite similar levels (11% up to 14%), suggesting that in this case, ZnO NWs enhances adsorption only, with almost no effect on photo catalysis. Furthermore, we can clearly see from both Figure 6c,d that saturation occurs at levels much below 100%. This saturation occurs after nearly 90 minutes, corresponding to the diffusion time τ at this scale of the gas cell. This can be ascribed to a saturation of physisorption, which is related to the limited specific surface area and hence to the limited sites for physisorption to occur.

## 4. Conclusions

We monitored *in situ* the depollution process of VOCs such as acetone and toluene in the presence of a ZnO-NWs array (NWA). The amount of pollutant gases was continuously monitored using a MEMS-FTIR spectrometer operating in the mid-infrared region, which appears as a very promising tool for monitoring air purification. An elimination ratio of 63% in 3 hours was achieved for toluene, while it was only 14% for acetone. The presence of the ZnO-NWs appears to be crucial in the case of acetone removal since almost no elimination (only 3%) was obtained in the absence of the ZnO-NWs. On the contrary, the ZnO-NWs were not as crucial for the elimination of the toluene; however, they enhance significantly the removal ratio. Our results obtained on ZnO-NWs can be compared to other nanostructured photocatalytic materials such as TiO_2_, α-Fe_2_O_3_/In_2_O and Ti_x_Zr_1-x_O_2_, for which the elimination ratio was reported to be 10-20% after 6 hours of purification [32,33,34]. It is also apparent from our results that in both cases, using UV light or not, due to their rather high specific area, ZnO NWs produces an enhancement in the purification for both mechanisms of adsorption and the photocatalysis reaction.

## References

[1] Ojala S., Pitkäaho S., Laitinen T., Koivikko N.N., Brahmi R., Gaálová J., Matejova L., Kucherov A., Päivärinta S., Hirschmann C. (2011). Catalysis in VOC abatement. Top. Catal..

[2] Burn J., Henk J., Bloemen T. (1993). Chemistry and Analysis of Volatile Organic Compounds in the Environment.

[3] Zhao J., Yang X. (2003). Photocatalytic oxidation for indoor air purification: A literature review. Build. Environ..

[4] Liao Q., Zhang Z., Zhang X., Mohr M., Zhang Y., Fecht H.-J. (2014). Flexible piezoelectric nanogenerators based on a fiber/ZnO nanowires/paper hybrid structure for energy harvesting. Nano Res..

[5] Kumar B., Kim S.-W. (2012). Energy harvesting based on semiconducting piezoelectric ZnO nanostructures. Nano Energy.

[6] Leschkies K.S., Divakar R., Basu J., Enache-Pommer E., Boercker J.E., Carter C.B., Kortshagen U.R., Norris D.J., Aydil E.S. (2007). Photosensitization of ZnO nanowires with CdSe quantum dots for photovoltaic devices. Nano Lett..

[7] Olson D.C., Piris J., Collins R.T., Shaheen S.E., Ginley D.S. (2006). Hybrid photovoltaic devices of polymer and ZnO nanofiber composites. Thin Solid Films.

[8] Liu K., Sakurai M., Aono M. (2010). ZnO-based ultraviolet photodetectors. Sensors.

[9] Soci C., Zhang A., Xiang B., Dayeh S.A., Aplin D.P.R., Park J., Bao X.Y., Lo Y.-H., Wang D. (2007). ZnO nanowire UV photodetectors with high internal gain. Nano Lett..

[10] Gong H., Hu J.Q., Wang J.H., Ong C.H., Zhu F.R. (2006). Nano-crystalline Cu-doped ZnO thin film gas sensor for CO. Sens. Actuators B Chem..

[11] Shishiyanu S.T., Shishiyanu T.S., Lupan O.I. (2005). Sensing characteristics of tin-doped ZnO thin films as NO2 gas sensor. Sens. Actuators B Chem..

[12] Azzouz I., Habba Y.G., Capochichi-Gnambodoe M., Marty F., Vial J., Leprince-Wang Y., Bourouina T. (2018). Zinc oxide nano-enabled microfluidic reactor for water purification and its applicability to volatile organic compounds. Microsyst. Nanoeng..

[13] Comini E., Baratto C., Faglia G., Ferroni M., Sberveglieri G. (2007). Single crystal ZnO nanowires as optical and conductometric chemical sensor. J. Phys. D. Appl. Phys..

[14] Habba Y.G., Capochichi-Gnambodoe M., Serairi L., Leprince-Wang Y. (2016). Enhanced photocatalytic activity of ZnO nanostructure for water purification. Phys. Status Solidi.

[15] Capochichi-Gnambodoe M., Habba Y.G., Leprince-Wang Y. (2016). A comparative study of the gas sensing properties of hierarchical ZnO nanostructures. Phys. Status Solidi.

[16] Kim I.-D., Rothschild A., Tuller H.L. (2013). Advances and new directions in gas-sensing devices. Acta Mater..

[17] Ma T., Guo M., Zhang M., Zhang Y., Wang X. (2007). Density-controlled hydrothermal growth of well-aligned ZnO nanorod arrays. Nanotechnology.

[18] Chevalier-César C., Capochichi-Gnambodoe M., Leprince-Wang Y. (2014). Growth mechanism studies of ZnO nanowire arrays via hydrothermal method. Appl. Phys. A.

[19] Colomb A., Yassaa N., Williams J., Peeken I., Lochte K. (2008). Screening volatile organic compounds (VOCs) emissions from five marine phytoplankton species by head space gas chromatography/mass spectrometry (HS-GC/MS). J. Environ. Monit..

[20] Yokelson R.J., Bertschi I.T., Christian T.J., Hobbs P.V., Ward D.E., Hao W.M. (2003). Trace gas measurements in nascent, aged, and cloud-processed smoke from African savanna fires by airborne Fourier transform infrared spectroscopy (AFTIR). J. Geophys. Res. Atmos..

[21] Gao H., Xie L., Gong P., Wang H. (2018). Detection of Ethanol Using a Tunable Interband Cascade Laser at 3.345 $μ$m. Photonic Sens..

[22] Hirschmann C.B., Lehtinen J., Uotila J., Ojala S., Keiski R.L. (2013). Sub-ppb detection of formaldehyde with cantilever enhanced photoacoustic spectroscopy using quantum cascade laser source. Appl. Phys. B.

[23] Kuusela T., Kauppinen J. (2007). Photoacoustic gas analysis using interferometric cantilever microphone. Appl. Spectrosc. Rev..

[24] Glière A., Rouxel J., Brun M., Parvitte B., Zéninari V., Nicoletti S. (2014). Challenges in the design and fabrication of a lab-on-a-chip photoacoustic gas sensor. Sensors.

[25] Eltagoury Y.M., Sabry Y.M., Khalil D.A. (2019). All-Silicon Double-Cavity Fourier-Transform Infrared Spectrometer On-Chip. Adv. Mater. Technol..

[26] Erfan M., Sabry Y.M., Sakr M., Mortada B., Medhat M., Khalil D. (2016). On-Chip Micro-Electro-Mechanical System Fourier Transform Infrared (MEMS FT-IR) Spectrometer-Based Gas Sensing. Appl. Spectrosc..

[27] Othman A.M., Kotb H.E., Sabry Y.M., Khalil D. (2019). EXPRESS: Micro--Electro--Mechanical Fourier Transform Infrared (MEMS FT-IR) Spectrometer under Modulated--Pulsed Light Source Excitation. Appl. Spectrosc..

[28] Kraft M., Kenda A., Sandner T., Schenk H. MEMS-based compact FT-Spectrometers-A platform for spectroscopic mid-infrared sensors. Proceedings of the Sensors, 2008 IEEE.

[29] Briand D., Manzardo O., de Rooij N.F., Hildenbrand J., Wollenstein J. Gas detection using a micromachined FTIR spectrometer. Proceedings of the Sensors, 2007 IEEE.

[30] Lammel G., Schweizer S., Renaud P. MEMS infrared gas spectrometer based on a porous silicon tunable filter. Proceedings of the Technical Digest. MEMS 2001. 14th IEEE International Conference on Micro Electro Mechanical Systems (Cat. No. 01CH37090).

[31] Gerguis J.O., Sabry Y.M., Omran H., Khalil D. (2019). Spectroscopic Gas Sensing Based on a MEMS-SOA Swept Fiber Laser Source. J. Light. Technol..

[32] Liu J., Li Y., Ke J., Wang S., Wang L., Xiao H. (2018). Black NiO-TiO2 nanorods for solar photocatalysis: Recognition of electronic structure and reaction mechanism. Appl. Catal. B Environ..

[33] Zhang F., Li X., Zhao Q., Zhang Q., Tadé M., Liu S. (2015). Fabrication of α-Fe2O3/In2O3 composite hollow microspheres: A novel hybrid photocatalyst for toluene degradation under visible light. J. Colloid Interface Sci..

[34] Liu B., Li X., Zhao Q., Ke J., Liu J., Liu S., Tadé M. (2015). Photocatalytic degradation of gaseous toluene with multiphase TixZr1- xO2 synthesized via co-precipitation route. J. Colloid Interface Sci..

